# Asparagus officinalis Exhibits Anti-Tumorigenic and Anti-Metastatic Effects in Ovarian Cancer

**DOI:** 10.3389/fonc.2021.688461

**Published:** 2021-07-14

**Authors:** Guangxu Xu, Weimin Kong, Ziwei Fang, Yali Fan, Yajie Yin, Stephanie A. Sullivan, Arthur-Quan Tran, Leslie H. Clark, Wenchuan Sun, Tianran Hao, Luyu Zhao, Chunxiao Zhou, Victoria L. Bae-Jump

**Affiliations:** ^1^ Department of Gynecology, Fengxian Hospital, Southern Medical University, Shanghai, China; ^2^ Division of Gynecologic Oncology, University of North Carolina at Chapel Hill, Chapel Hill, NC, United States; ^3^ Department of Gynecologic Oncology, Beijing Obstetrics and Gynecology Hospital, Capital Medical University, Beijing, China; ^4^ Department of Obstetrics, Beijing Obstetrics and Gynecology Hospital, Capital Medical University, Beijing, China; ^5^ Shandong Juxinyuan Agricultural Technology Co, LTD., Heze, China; ^6^ Lineberger Comprehensive Cancer Center, University of North Carolina at Chapel Hill, Chapel Hill, NC, United States

**Keywords:** Asparagus officinalis, cell proliferation, apoptosis, invasion, ovarian cancer

## Abstract

Ovarian cancer is one of the leading causes of female cancer death. Emerging evidence suggests that many dietary natural products have anti-tumorigenic activity, including that of asparagus officinalis. The current study aimed to assess the anti-tumorigenic and anti-metastatic effects of asparagus officinalis on serous ovarian cancer cell lines and a transgenic mouse model of high grade serous ovarian cancer. Asparagus officinalis decreased cellular viability, caused cell cycle G1 phase arrest and induced apoptosis in the OVCAR5 and SKOV3 cells. Induction of apoptosis and inhibition of cell proliferation was rescued by the pan-caspase inhibitor, Z-VAD-FMK, implying that its cytotoxic effects were mainly dependent on caspase pathways. Asparagus officinalis increased levels of ROS and decreased mitochondrial membrane potential with corresponding increases in PERK, Bip, Calnexin PDI and ATF4 in both cell lines. Treatment with asparagus officinalis also reduced ability of adhesion and invasion through epithelial–mesenchymal transition and reduction of VEGF expression. The combination of Asparagus officinalis with paclitaxel had synergistic anti-proliferative activity. Furthermore, Asparagus officinalis significantly inhibited tumor growth and reduced serum VEGF in a genetically engineered mouse model of ovarian cancer under obese and lean conditions, accompanied with a decrease in the expression of Ki67, VEGF and phosphorylated S6, and in an increase in phosphorylation of AMPK in the ovarian tumor tissues. Overall, our data provide a pre-clinical rationale for asparagus officinalis in the prevention and treatment of ovarian cancer as a novel natural product.

## Introduction

Ovarian cancer (OC) is the most lethal gynecologic malignancy and ranks fifth in overall cancer deaths in women, with an anticipated 21,410 new cases and 13,770 deaths anticipated for 2021 in the United States (1). Due to the lack of an effective method for the early detection of this disease and nonspecific symptoms at early stages, greater than 75% of ovarian cancer cases go undetected until an advanced stage, with an overall survival rate of less than 40% at 5 years (2, 3). The mainstay of treatment for ovarian cancer is tumor debulking surgery followed by platinum and paclitaxel chemotherapy. Bevazizumab and PARP inhibitors are now additional drugs in advanced ovarian cancer (4). Patients typically have high initial response rates, but the majority of patients are ultimately faced with a recurrence and platinum resistance despite significant advances in treatment strategies (3, 5). Thus, effective management of recurrence and chemo-resistance remains a significant challenge, and novel therapies including targeted therapy need to be developed to improve outcomes.

Asparagus officinalis (ASP) is a perennial vegetable with remarkable anti-fungal, anti-hepatotoxic, cytotoxic, anti-mutagenic, anti-inflammatory and diuretic properties (6). The roots and buds of ASP are rich in many bioactive phytochemicals including oligosaccharides, steroidal saponins, amino acid derivatives and essential minerals, which has been used for treatment of various chronic inflammation diseases and cancers in Chinese traditional medicine (6–8). Polysaccharides, steroidal saponins and flavonoids extracted from ASP have been reported as main bioactive constituents that might have anti-tumor properties (9). A large prospective epidemiological study showed that high consumption of composite vegetables, including ASP, was inversely associated with risk of hepatocellular carcinoma (10). ASP polysaccharide reduced cell proliferation of hepatocellular carcinoma cells and tumor growth in HepG2 cell xenograft tumors in nude mice through induction of apoptosis (11). Saponins extracted from ASP exerted potential inhibitory activity on cell growth and invasion in various cancer cells (12). Bousserouel et al. recently found that white ASP extracts induced cell death and activated TRAIL DR4/DR5 apoptosis pathways in the SW480 colon carcinoma cells and their derived metastatic cells (SW620). Feeding wistar rats with fresh ASP in drinking water caused a 50% reduction in the number of preneoplastic lesions at the surface of the colon in a rat model of colon carcinogenesis using azoxymethane (AOM)-initiated colon cancer (13). These results suggest that ASP has potential anti-tumorigenic and anti-metastatic activity against a wide range of cancers in preclinical models. Our current study aimed to evaluate the possible anti-tumorigenic and anti-metastatic effects of the ASP extract on cell proliferation, apoptosis, cell cycle progression, cell stress, invasion and tumor growth in human serous ovarian cancer cell lines and a transgenic mouse model of high grade serous ovarian cancer. This is the first study to investigate the inhibitory effect of ASP on tumor growth and invasion in ovarian cancer.

## Materials and Methods

### Cell Culture and Reagents

The OVCAR5, SKOV3, OVCAR3, OV433, IGROV-1 and Hey cell lines were utilized in this study. The OVCAR5, OVCAR3, OV433 and SKOV3 cells were maintained in DMEM/F12 medium with 10% bovine serum. The IGROV-1 cells were grown in RPMI 1640 medium supplemented with 10% bovine serum. The Hey cells were maintained in RPMI 1640 medium supplemented with 5% bovine serum. All media included 100 units/ml penicillin and 100 microgram/ml streptomycin. Cells were cultured at 37°C in 5% CO2 humidified incubators. All antibodies used were from Cell Signaling (Danvers, MA).

### Preparation of ASP Extract

The extract from shoots of ASP was supplied by the Shandong Juxinyuan Agricultural Technology Co, LTD and Shandong Shuoyi Biotechnology Co, LTD, P.R. China. ASP was grown in greenhouse without pesticides and chemical fertilizers, only organic matter or manure was applied to the plants during the growing season. If necessary, ASP wastes included broken spears, bottom cuts and other unmarketable parts were disposed and used as natural pesticides. Briefly, the crushed stems and shoots were washed in distilled water at 95°C and then extracted with squeezer equipment. After centrifuge to remove debris fragments, the crude extract was concentrated by vacuum and then extracted using a water bath. The final extract contains 70% soluble substances and is then sterilized under pasteurization. Additionally, 11 pesticide ingredients are tested by local government agency. The results showed that no pesticide components were detected in the extracts (**Supplemental Tables 1** and **2**).

### Cell Proliferation Assay

The OVCAR5 and SKOV3 cells (4000 cells/well) were plated in 96 well plates for 24 hours. The cells were then incubated with different doses of ASP for 72 hours. Add 5ul of MTT (5mg/ml, Sigma) to each well for 1 hour. 100 ul dimethyl sulfoxide (DMSO) was added into each well to stop the reaction. The absorbance at 595 nm was determined in each well with a Tecan plate reader. The effect of ASP on cell inhibition was calculated as a percentage of control cell growth. These experiments were repeated three times to confirm consistency of results.

### Colony Formation Assay

The OVCAR5 and SKOV3 cells were seeded in 6-well plates (200 cells/well) in their standard growth media overnight, and then were treated with ASP for 24 hours. The cells were continually cultured for 12 days with media changes every third or fourth day. Subsequently, cells were fixed with 4% paraformaldehyde and stained with 0.1% crystal violet. The number of colonies were counted under the microscope. Each experiment was repeated three times for consistency of results.

### Apoptosis Assay

The expression of Annexin V (Annexin V FITC kit, Biolegend, San Diego, CA) was detected by Cellometer (Nexcelom, Lawrence, MA). 2.5 x 10^5^/ml cells were seeded into six well plates. After 24 hours, the cells were treated with various doses of ASP for 12-18 hours. The cells were harvested and suspended in binding buffer. Added Annexin V to the binder buffer and placed in the dark for 15 minutes. The Cellometer was then used to measure the samples, and FCS4 express software was used to interpret the data. All experiments were repeated three times.

### Cleaved Caspase 3, 8 and 9 Assays

The OVCAR5 and SKOV3 cells (2.5 × 10^5^ cells/well) were plated in 6-well plates for 24 hours and then the cells were treated with ASP at the indicated concentrations for 14 hours. Cells were washed with PBS and added lysis buffer into each well. Reaction buffer contained caspase 3, 8 and 9 substrates was added to lysis buffer in a new black 96-well plate at 37°C for 20-30 minutes. The fluorescence intensity (excitation/emission=465/540) for Caspase 3, 8 and 9 activities were recorded using a Tecan plate reader (14). These assays were repeated three times

### Cell Cycle Progression Assay

Cell cycle progression was assessed and measured by Cellometer. 2.5 x 10^5^/ml cells were plated on 6-well plates, incubated for 24 hours and then treated with various doses of ASP for 48 hours. The cells were harvested and washed with PBS. 90% methanol was added to the cells and the cells were maintained overnight at -20°C. The cells were re-suspended in 50 ug RNase A solution with 10 mM Propidium Iodide and 0.05% Triton X-100 followed by 30 minutes of incubation. The samples were then assessed by Cellometer. FCS4 express software was used to analyze the data. All experiments were repeated twice for consistency of results.

### Adhesion Assay

Laminin-1 was use to coat each well of 96 well plates and then incubated for 2 hours. OVCAR5 and SKOV3 (1.2 x 10^5^ per ml) cells were cultured with varying concentrations of ASP for 2 hours and then the cells were fixed with the addition of 5% glutaraldehyde for 30 minutes. Adherent cells were stained with 0.1% crystal violet (100 ul) for 20-30 minutes. The cells were washed with water and acetic acid (100 ul) was added to each well. Shaking plate for 5 minutes. The absorbance was measured at 570 nm using a microplate reader. Each experiment was repeated three times.

### Invasion Assay

Invasion ability was determined using Transwell Migration Assay system (Thermo Fisher Scientific) according to the manufacturer’s protocol. OVCAR5 and SKOV3 cells were cultured with serum free media for 12 hours and then seeded in the upper chambers of the wells in a 96 well plate. The lower chamber was added with regular media containing different doses of ASP for 4 hours. After washing lower chambers with PBS, 100 uL calcein AM solution was added to the lower chambers for 30-60 minutes. The lower chamber plate was measured by Tecan plate reader for reading fluorescence at EX/EM 485/520 nm. Each experiment was repeated three times.

### Reactive Oxygen Species Assay

The production alterations of reactive oxygen species caused by ASP was detected *via* DCFH-DA assay. In brief, the OVCAR5 and SKOV3 (6500 cells/well) were seeded in black 96-well plates and allowed to incubate for 24 hours. Then the cells were subjected to ASP at indicated concentrations for 8 hours. After treatment, DCFH-DA (20 μM) in phenol-red-free regular media was then applied and kept for 30 minutes. The analysis was conducted by Tecan plate reader in a setting of excitation/emission wavelength of 485/530 nm separately. The experiments in triplicate were carried out to make the results sense.

### Mitochondrial Membrane Potential Assays

JC-1 assay was used to detect ASP induced mitochondrial membrane potential (15, 16). The OVCAR5 and SKOV3 cells (6,000 cells/well) were incubated into 96-well plates overnight and treated with ASP at indicated doses for 8 hours. The media were removed and 100ul of warmed PBS with 2 uM JC-1 was added to each well for 30 minutes at 37°C. The plate reader (Tecan) monitored the fluorescence intensity at the excitation/emission wavelength of 530/580 nm. Each experiment was repeated three times.

### Western Immunoblotting

The OVCAR5 and SKOV3 cell lines were treated for 8 to 24 hours with different concentrations of ASP in their appropriate medium. RIPA lysis buffer was used to lyse the cells for 30 minutes on ice. The protein concentration was quantified using a bicinchoninic acid (BCA) assay (Bio-Rad, Hercules, CA). 30 µg of protein was loaded per lane and separated using 12% sodium dodecyl sulfate-polyacrylamide gel electrophoresis (SDS-PAGE). The proteins were transferred onto PVDF membranes. The membrane was incubated with a primary antibody at 4°C overnight. After washing with TBST buffer, the membranes were incubated with the secondary antibodies for 1 hours at room temperature. Antibody binding was detected using SuperSignal™ West Pico on the ChemiDoc™ Image System (Bio-Rad). Each experiment was repeated multiple times to assess for consistency of results

### Detection of VEGF Concentrations in Mice Serum

Mouse VEGF ELISA kit (R&D Systems, Minneapolis, MN) was used to detect the concentrations of VEGF in serum according to the manufacturer’s instructions.

### Transgenic Mouse Model of High-Grade Serous Ovarian Cancer

The $\text{K}18-\text{g}{\text{T}}_{121}^{+/-}$; p53^fl/fl^; Brca1^fl/fl^ (KpB) mouse model has been described previously (17, 18). Animal protocol was approved by the Institutional Animal Care and Use Committee (IACUC) in the University of North Carolina at Chapel Hill. In the implementation of animal experiments, we strictly follow the protocol (19-141). Ad5-CMV-Cre (Transfer Vector Core, University of Iowa) was injected into the left ovarian bursa cavity of KpB mice at 6–8 weeks age. The mice were randomly divided into four groups (15 mice per group), including a control group and two ASP treatment groups. In the treatment groups, the mice were treated with 200 or 800 mg/kg daily by oral garage for 4 weeks. The concentrated extract without ASP was used to treat control mice.

In order to explore the effect of ASP on tumor growth in KpB mice under obese and lean conditions, the mice were given a LFD (Low fat diet) or an HFD (High fat diet, Research diet, New Brunswick, NJ) at the age of 3 weeks and divided four groups: HFD control, HFD +ASP, LFD control and LFD+ASP. When ovarian tumor in diameter reached 0.1-0.2 cm, the mice were treated with ASP (200 mg/kg, for 4 weeks, daily) by oral garage. During the treatment, the ovarian tumor was checked weekly using palpation until tumors had grown to a size amenable to caliper measurement. Mice were euthanized after ASP treatment. Ovarian tumors and blood samples were harvested and stored at −80°C until use. Tumor volume was calculated using the following: (width^2^ × length)/2.

### Immunohistochemical (IHC) Analysis

The paraffin-embedded tissue sections were continuously cut at 5 μm from ovarian cancer tissues of KpB mice and dewaxed. Citrate buffer was used for antigen retrieval. The sections were incubated with primary antibodies with Ki67, phosphorylated-AMPK, phosphorylated-S6, and VEGF overnight at 4°C. After application of secondary antibodies, sections were applied with an ABC Substrate System (Vector Labs, Burlingame, CA) for the color reaction and with Mayer’s hematoxylin for counterstaining. The sections were scanned by Motic and scored by ImagePro software (Rockville, MD).

### Statistical Analysis

Data are presented as a mean ± the standard error of the mean. Statistical tests and graphs were generated using GraphPad Prism 8 software. An unpaired Student’s t test was used for comparisons between groups. P values <0.05 were considered statistically significant.

## Results

### ASP Inhibited Cell Proliferation

**Figure 1:**
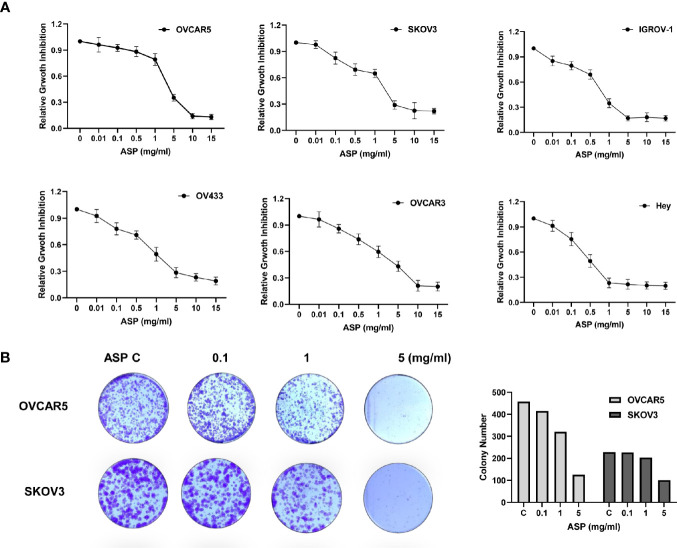
ASP inhibited OC cell proliferation and colony formation. The OVCAR5, OVCAR3, Hey, OV433, IGOV-1 and SKOV3 cell lines were treated with different concentrations of ASP in 96-well plates for 72 hours. Cell proliferation was assessed by MTT assay. ASP suppressed cell proliferation in a dose-dependent manner in OC cells **(A)**. The effect of ASP on the long-term growth of the OVCAR5 and SKOV3 cell lines was assessed through a colony-forming assay. ASP inhibited colony formation in both cell lines after 12 days of treatment **(B)**. All data are presented as mean ± SE of triplicate samples and are representative of three independent experiments.

The OC cell lines OV433, OVCAR3, IGROV-1, Hey, OVCAR5 and SKOV3 were exposed for 72 hours to an extract of ASP at concentrations varying from 0.01 to 15 mg/ml. The MTT assay showed that with increasing concentrations of ASP, cell viability decreased in a dose−dependent manner, compared to the control cells in all six OC cell lines (**Figure 1A**). The mean IC50 values of ASP were 3.5 mg/ml for OVCAR5, 0.75 mg/ml for IGROV-1, 0.51 mg/ml for Hey, 4.9 mg/ml for OVCAR3, 1 mg/ml for OV433 and 4.2 mg/ml for SKOV3. Given that the colony formation assay is a well-established *in vitro* method for testing the proliferative capacity of treated cells, we evaluated the long-term effect of ASP on colony formation in the OC cell lines. Our results revealed the significantly decreased colony-forming capacity of OVCAR5 and SKOV3 cells by 71.3% and 56.2%, respectively, when exposed to 5mg/ml ASP for 24 hours and subsequent culture of the cells for 12 days, as compared to those not exposed to the ASP (**Figure 1B**). These results indicate that ASP has the ability to inhibit cell proliferation of OC *in vitro*.

### ASP Induced Apoptosis

**Figure 2:**
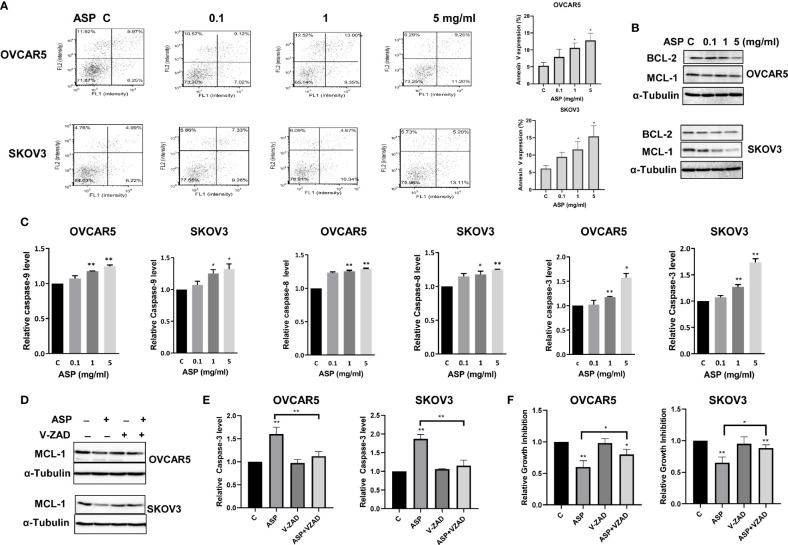
ASP prompted apoptosis in OC cells. ASP increased the expression of Annexin V in the OVCAR5 and SKOV3 cells in a dose dependent manner after 18 hours of treatment **(A)**. The cells were treated with the indicated concentration of ASP for 24 hours. Western blotting showed that ASP reduced the expression of BCL-XL and MCL-1 in both cells **(B)**. The activities of cleaved caspase 3, 8 and 9 for the cells treated with different concentrations of ASP were significantly increased after 14 hours of treatment **(C)**. OVCAR5 and SKOV3 cells were pretreated with VAD-FMK (10 µM) for 1 hour, followed by treatment with ASP for 18 and 48 hours. Pretreatment with Z-VAD-FMK partially blocked ASP’s effects on reducing MCL-1 expression and inducing cleaved caspase-3 activity in both cells **(D, E)**. Blocking caspase pathways by Z-VAD-FMK in combination with ASP significantly rescued cell viability **(F)**. All experiments were repeated three times. *p < 0.05, **p < 0.01.

To determine whether ASP could induce apoptosis in OC cells, an Annexin V assay was performed in the OVCAR5 and SKOV3 cells after 18 hours of treatment with different concentrations of ASP. **Figure 2A** illustrates the Annexin V assay results for both cell lines. Treatment with ASP increased annexin V expression in a dose dependent manner, along with decreasing protein expression of MCL-1 and BCL-XL in both cell lines (**Figure 2B**). To evaluate whether mitochondrial apoptotic pathways were involved in ASP-induced apoptosis in the OC cells, ELISA assays were used to detect the activities of cleaved caspase 3, 8 and 9 in both cell lines after 14 hours of treatment. ASP significantly increased the level of cleaved caspase 3, 8 and 9 in a dose dependent manner. ASP at 5mg/ml increased cleaved caspase 3 by 1.58 in the OVCAR5 cells and 1.74 times in the SKOV3 cells (**Figure 2C**). To further examine the role of ASP on mitochondrial apoptotic pathway, the OVCAR5 and SKOV3 cells were pre-treated with the caspase inhibitor Z-VAD-FMK (10 µM) for 1 hour, followed by treatment with ASP for 18 and 48 hours. Pre-treatment with Z-VAD-FMK partially blocked ASP’s effects on reducing MCL-1 expression and inducing cleaved caspase-3 activity (**Figures 2D, E**). Similarly, Z-VAD-FMK significantly rescued cell viability from ASP-mediated apoptosis in both cell lines (**Figure 2F**). These results suggest that ASP inhibits cell proliferation through induction of the mitochondrial apoptotic pathway in OC cells.

### ASP Induced Cell Cycle G1 Arrest

**Figure 3:**
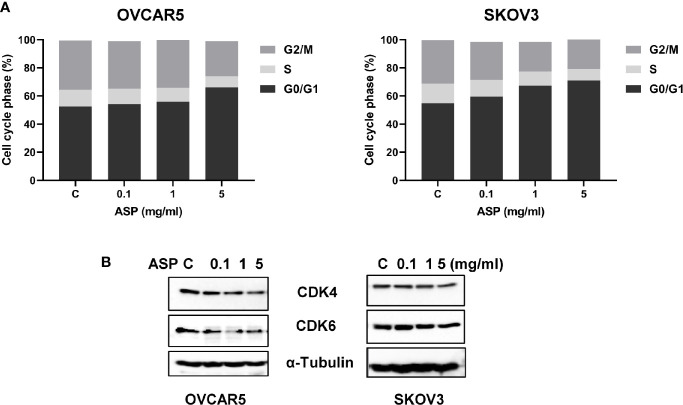
Induction of cell cycle G1 arrest by ASP is dose-dependent. The OVCAR5 and SKOV3 cells were incubated in the presence of the indicated doses of ASP for 48 hours. The cell cycle phase distribution was determined by Cellometer. ASP induced an arrest of cells at the G1 phase in a dose-dependent manner in both OVCAR5 and SKOV3 cells **(A)**. To evaluate expression of cell cycle related proteins, the OVCAR5 and SKOV3 cells were treated with ASP for 24 hours. Western blotting results showed that the expression of CDK4 and CDK6 proteins were decreased after treatment with ASP **(B)**. The results shown are one of three independent experiments.

To instigate whether ASP could affect cell cycle progression in OC cells, the cell cycle was evaluated by Cellometer in the OVCAR5 and SKOV3 after exposure to ASP. Both cell lines were treated with ASP at differing doses (0.1-5 mg/ml) for 48 hours. Cellometer results showed that ASP increased G0/G1 cell cycle phase and reduced S phase in a dose-dependent manner. G1 phase increased from 52.5% in control to 66.3% at a dose of 5 mg/ml in OVCAR5 cells and 54.2% to 71.6% in SKOV3 cells (**Figure 3A**). Moreover, western blotting results confirmed that ASP significantly decreased CDK4 and CDK6 expression after 24 hours of treatment (**Figure 3B**). These data suggest that inhibition of cell growth by ASP is also the results of cell cycle G1 arrest in OC cells.

### ASP Induced Cellular Stress

**Figure 4:**
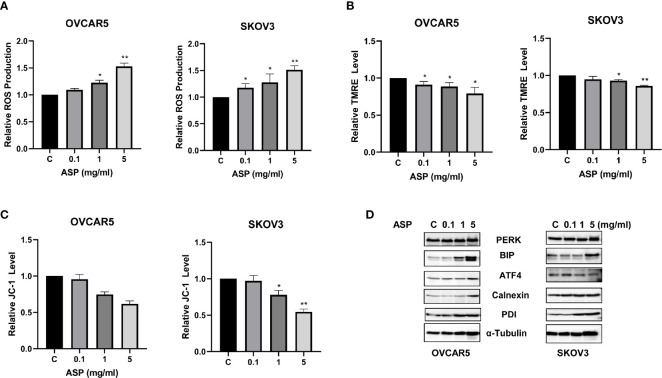
ASP induced cellular stress in OC cells. Effects of ASP on ROS, JC-1 and TMRE products were measured by ELISA assays. ASP significantly increased the levels of ROS and decreased JC-1 and TRME products in both cell lines compared to the control-treated cells after 8 hours of treatment **(A–C)**. The results from Western blotting analysis showed that ASP increased the expression of the cellular stress related proteins in both cell lines after treatment for 24 hours, including PERK, Bip, ATF4, Calnexin and PDI **(D)**. *p < 0.05; **p < 0.01.

Given the role of ROS as a mediator of apoptosis in cancer cells (19), we investigated whether the apoptosis induced by ASP was involved in ROS generation in OC cells. Cellular ROS productions were assessed using the ROS fluorescence indicator DCFH-DA assay. ASP treatment for 8 hours significantly increased ROS production in a dose-dependent manner in both cell lines. ASP increased ROS by 1.54 times in the OVCAR5 cells and 1.51 times in the SKOV3 cells at a dose of 5mg/ml compared to untreated cells (**Figure 4A**). To further characterize whether this increase in ROS is associated with mitochondrial function, the JC-1 and TMRE ELISA assays were used to detect alterations of mitochondrial membrane potential (ΔΨm) after ASP exposure. Both assays showed that ASP induced the loss of ΔΨm in a dose-dependent manner in both cell lines after 8 hours of treatment compared to control cells (**Figures 4B, C**). Furthermore, ASP regulated the expression of the endoplasmic reticulum (ER) stress-related proteins in the OVCAR5 and SKOV3 cells. Western immunoblotting showed that ASP up-regulated the protein expression of PERK, ATF4, PDI, BiP and Calnexin in both cell lines after 24 hours of treatment (**Figure 4D**). These results suggest that an increase in ROS production and loss of ΔΨm are also associated with the anti-proliferative effects of ASP in OC cells.

### ASP Inhibited Tumor Growth in KpB Mice

**Figure 5:**
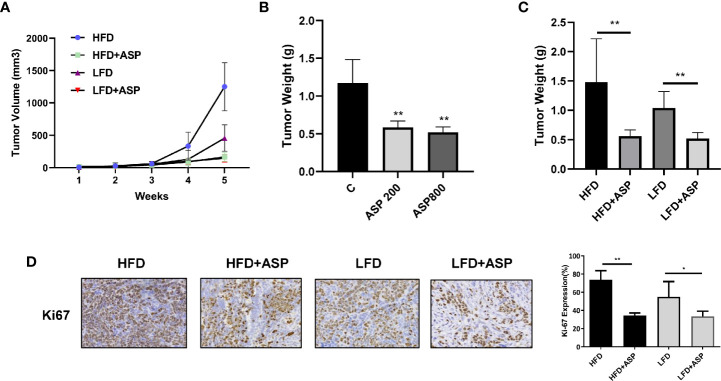
ASP inhibited tumor growth in a transgenic mouse model of OC (KpB). The KpB mice were treated with 200mg/kg or 800mg/kg ASP daily for 4 weeks after ovarian tumors reached 0.1 cm in diameter. Treatment with 200 mg/kg ASP inhibited tumor growth in KpB mice **(A)**. There was no significant difference in tumor weights after treatment with 200mg/kg *versus* 800mg/kg ASP **(B)**. The KpB mice were then fed either a HFD (obese) or LFD (lean) at 3 weeks age of birth. The HFD and LFD mice were treated with 200 mg/kg ASP daily for 4 weeks when their ovarian tumors reached 0.1 cm in diameter. ASP significantly reduced tumor weights in both the HFD and LFD groups as compared to the vehicle treated controls **(C)**. IHC results showed that ASP decreased Ki-67 expression in the ovarian tumor tissues in the obese and lean mice **(D)**. *p < 0.05; **p < 0.01.

To determine whether different doses of ASP affect tumor growth, KpB mice, a transgenic mouse model of high grade serous OC, were treated with 200mg/kg or 800 mg/kg ASP or vehicle daily through oral gavage (15 mice/per group). After 4 weeks of treatment, ovarian tumor weights were decreased in mice treated with 200mg/kg or 800mg/kg ASP compared with mice in the vehicle group (**Figures 5A, B**). ASP decreased tumor weight by 51% in the 200 mg/kg group and by 56% in the 800mg/kg group. There was no statistical difference in the anti-tumorigenic effects between the 200 and 800 mg/kg treatment groups. The mice showed tolerance to ASP at 200 or 800 mg/kg and maintained normal activities. No changes in body weight or blood glucose levels were detected in ASP-treated mice over 4 weeks.

Given that we previously confirmed that obesity significantly promoted tumor growth in the KpB mice (17, 20, 21) and that ASP is a dietary product, we next investigated whether ASP reduces tumor growth under both obese and lean conditions. KpB mice were fed the low fat diet (LFD; lean) or high fat diet (HFD; obese) at 3 weeks of age and divided into four groups (LFD and HFD controls, LFD +ASP and HFD+ASP groups, 15 mice/group). The average animal body weight was 25.9 g in the LFD group and 39.6g in the HFD group at the beginning of the ASP treatment. The mice were administrated ASP daily by oral gavage (200mg/kg) for 4 weeks once tumors reached 0.1-0.2 cm in diameter. At the end of the treatment, a HFD (control) significantly increased overall tumor weight compared to LFD control mice (1.48 g versus 1.01 g). ASP-induced decreases in tumor weight in the HFD group were significantly greater than in LFD mice (63% versus 50%, p<0.05), indicating that anti-tumorigenic efficacy of ASP may be related to obesity status (**Figure 5C**). In order to evaluate effect of ASP on tumor cell proliferation after ASP treatment, the expression of ki67 was detected by IHC analysis. ASP inhibited the expression of Ki-67 in the HFD mice by 39% and in the LFD by 21% compared to each control group (**Figure 5D**).

### ASP Inhibited Adhesion and Invasion

**Figure 6:**
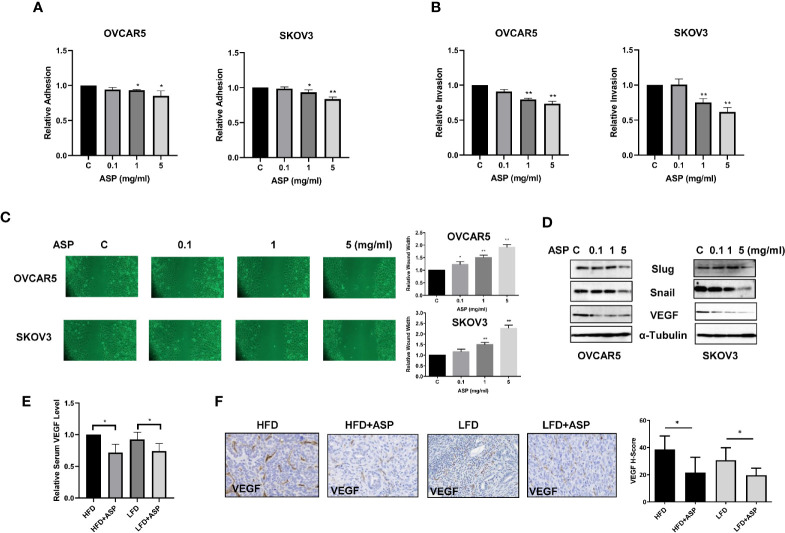
ASP inhibited adhesion and invasion. The OVCAR5 and SKOV3 were treated with ASP at a range of doses from 0.1−5 mg/ml. laminin-1 assay showed that treatment OVCAR5 and SKOV3 cells with ASP for 2 hours significantly reduced cell adhesion **(A)**.Invasion was determined by transwell assay after 4 hours of treatment with ASP. ASP significantly reduced cell invasion in a dose-dependent manner in both cells **(B)**. Migration was assessed by wound healing assay after treatment with ASP for 48 hours. The results showed that ASP reduced OC cell migration **(C)**. Western blotting found that ASP reduced the expression of VEGF, Snail and Slug in both cell lines **(D)**. The obese and lean KpB mice were treated with 200 mg/kg ASP for 4 weeks. ASP decreased serum VEGF products in both obese and lean mice **(E)**. IHC results showed that ASP reduced VEGF expression in both obese and lean mice **(F)**. *p < 0.05, **p < 0.01.

Given that ASP previously exhibited anti-invasion activity in pancreatic, breast and colon cancer cells (12), adhesion, invasion and wound healing assays were used to detect the effects of ASP on adhesion and invasion the OC cell lines. The OVCAR5 and SKOV3 cells were treated with ASP for 2 hours in 96 well plate coated with laminin-1. A significant decrease in cell adhesion was observed in ASP treatment cells (**Figure 6A**). Cell invasion was detected using a transwell invasion assay with a matrigel-coated insert. OVCAR5 and SKOV3 cells were cultured in the upper chambers of the transwell and incubated with different concentrations of ASP for 4 hours. Cellular invasion was decreased by 27.4% in the OVCAR5 cells and 38.4% in the SKOV3 cells, respectively (**Figure 6B**). Wound healing was monitored at 0, 24 and 48 hours, and the results showed that ASP reduced cell migration at 24 and 48 hours of treatment compared to control groups (**Figure 6C**). To determine the effect of ASP on epithelial-to-mesenchymal transition (EMT) and angiogenesis in OC cells, OVCAR5 and SKOV3 cells were treated with ASP for 24 hours. Western blotting results showed that ASP reduced the expression of slug, snail and VEGF (**Figure 6D**).

To examine whether ASP reduced VEGF levels in serum and tumor tissue of KpB mice, serum VEGF was measured by ELISA assay. ASP significantly decreased VEGF production by 28.3% in the HFD group and 24.1% in the LFD group compared to control mice (**Figure 6E**). In the ovarian tissues of KpB mice fed a HFD, VEGF expression was increased by 8% compared to the mice fed a LFD. IHC results showed that ASP treatment reduced the expression of VEGF in ovarian tumors by 16% in the HFD group and 11% in the LFD group (**Figure 6F**). Overall, these results suggest that ASP has potential to inhibit invasion in OC cells and KpB mouse model of OC

### ASP Activated AMPK and Inhibited mTOR Pathways

**Figure 7:**
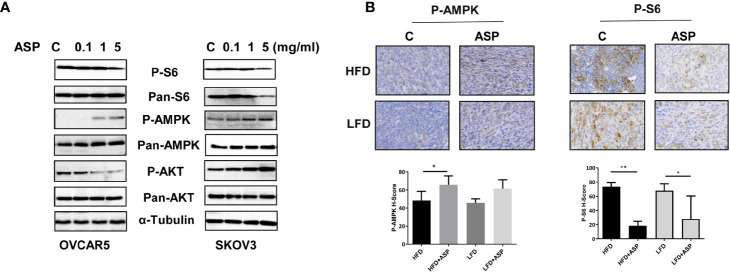
ASP activated AMPK and inhibited the AKT/mTOR pathways. The OVCAR5 and SKOV3 ovarian cell lines were treated with ASP at indicated doses for 24 hours. The expression of phosphorylated AMPK, Akt and S6 was analyzed by western blotting. ASP increased the expression of phosphorylated AMPK and decreased the expression of phosphorylated S6 in both cell lines. ASP decreased the expression of phosphorylated AKT in the OVCAR5 cells and increased phosphorylated AKT expression in the SKOV3 cells **(A)**. IHC results showed that ASP increased phosphorylated AMPK and decreased phosphorylated S6 expression in the ovarian cancer tissues of obese and lean mice **(B)**. *p < 0.05; **p < 0.01.

To examine whether the AMPK/mTOR pathway plays a role in the anti-tumorigenic activity of ASP, the OVCAR5 and SKOV3 cells were treated with different concentrations of ASP for 24 hours, and western blotting was used to detect changes in the AMPK/mTOR pathway using phosphorylated AKT, AMPK and S6 antibodies. We found that phosphorylated AMPK expression was upregulated and phosphorylated S6 was downregulated in both cell lines following ASP treatment. However, ASP increased phosphorylated AKT expression in the SKOV3 cells and decreased expression of phosphorylated AKT in the OVCAR5 cells, suggesting that ASP inhibited mTOR signaling through a negative feedback loop in SKOV3 cells (**Figure 7A**). To further investigate the role of ASP and the AMPK/mTOR pathway *in vivo*, the expression of phosphorylated S6 and AMPK was evaluated by IHC in the ovarian tumors of KpB mice. As expected, ASP treatment significantly increased the H-Score of phosphorylated AMPK from 48 to 65 in the HFD group and from 45 to 61 in the LFD group. Moreover, treatment with ASP also reduced the H-Score of phosphorylated S6 in the HFD and LFD groups compared with the control mice, indicating that ASP inhibited tumor growth through the AMPK/mTOR pathway *in vitro* and *in vivo* (**Figure 7B**).

### ASP in Combination With Paclitaxel Synergistically Inhibits the Cell Viability

**Figure 8:**
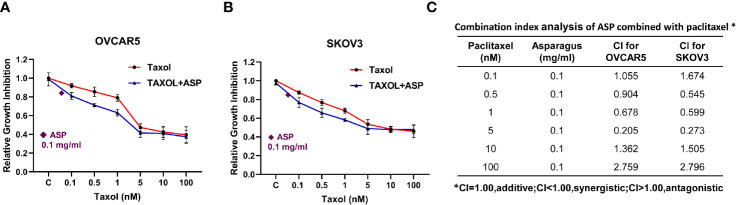
ASP increased sensitivity to paclitaxel in OC cells. The OVCAR5 and SKOV3 ovarian cell lines were treated with 0.1 mg/ml ASP combined with different concentrations of paclitaxel for 48 hours. Cell proliferation was assessed by MTT assay. ASP combined with paclitaxel (0.1-5 nM) caused a synergistic cytotoxic effect in both cells **(A, B)**. The combination index (CI) was calculated using CompuSyn software **(C)**. *CI < 1: synergism, CI = 1: additive effect, and CI > 1: antagonism.

To evaluate the synergistic effect of ASP on paclitaxel in OC cells, the OVCAR5 and SKOV3 cell lines were treated with varying concentrations of paclitaxel and then in combination with different concentrations of ASP for 72 hours. Cell viability was evaluated using the MTT assay. Relative cell viability was decreased in a dose-dependent manner following treatment with paclitaxel or ASP. When ASP concentrations between 1 and 5 mg/ml were combined with different concentrations of paclitaxel, additive or antagonistic effects were observed in both cell lines. In contrast, 0.5 mg/ml or 0.1 mg/ml of ASP combined with paclitaxel (0.1-5 nM) led to a greater inhibition of cell viability than those of ASP or paclitaxel alone (**Figures 8A, B**). To quantify the response of OVCAR5 and SKOV3 cells to the combination of ASP and paclitaxel, the combination index (CI) was calculated using CompuSyn software. The results showed that 0.1 mg/ml or 0.5 mg/ml (data not shown) of ASP combined with paclitaxel (0.1-5 nM) caused a synergistic cytotoxic effect (CI<1) in both cell lines (**Figure 8C**).

## Discussion

There is growing evidence that selected dietary products such as fruits and vegetables as part of complementary medicine may have promise in the prevention and treatment of cancers (22). ASP has been used as a traditional herbal medicine to treat diabetes, fever, kidney disease, inflammation and cancers in both European and Asian countries (10, 22–24). Recent research reports have suggested that ASP has the potential to be an anti-neoplastic agent because it exhibits a wide range of anti-tumorigenic activity through targeting multiple cell signaling and metabolic pathways *in vitro* and *in vivo* (11–13, 18, 24–26). In this study, we used the extract of ASP to investigate its effects on cell proliferation, cellular stress, invasion and tumor growth in serous OC cell lines and a transgenic mouse model of high grade serous OC. ASP was found to significantly inhibit cell proliferation, increase cellular stress, induce cell cycle G1 arrest and apoptosis, and decrease invasion in OC cells. Importantly, ASP inhibited ovarian tumor growth along with decreasing Ki67, VEGF and phosphorylated-S6 as well as increasing phosphorylated-AMPK expression in ovarian tumors of both obese and lean KpB mice, a model of high grade serous OC. Furthermore, low concentrations of ASP synergistically increased sensitivity to paclitaxel in OC cells. Our results are the first to delineate that ASP exerts anti-proliferative and anti-metastatic effects by targeting the AMPK/mTOR pathway in OC through our *in vitro* and *in vivo* studies.

ASP is a popular and healthy vegetable that is rich in vitamins, minerals and amino acids and fiber (7). Despite its promise in the treatment of various diseases, there is relatively little knowledge about the biochemistry of ASP and its numerous pharmacological activities and mechanisms. The phytochemical investigation of ASP with the aim of isolating and identifying active compounds has led to the isolation and identification of steroidal saponins, saccharides, flavonoids, phenolic compounds, acetylenic compounds and sulfur-containing compounds, among others, from this vegetable (12, 23, 24, 27, 28). The majority of species extracted from ASP have elicited a wide range of ant-tumorigenic activities in different cancer cells, such as inhibiting cell proliferation, inducing apoptosis/cell cycle arrest and inhibiting invasion *via* multiple signaling pathways (12, 13, 25, 29). Although different sections of ASP contain different concentrations of the active compounds (7), the extracts from different parts of ASP seem to exhibit similar anti-tumorigenic activity against different types of cancer cells. Saponins extracted from different parts of ASP show similar inhibition of cell growth in different cancer cell lines (12, 24, 30). The extracts from ASP shoots reduced cell proliferation of colon cancer *via* activating the TRAIL apoptotic death pathway and inhibited colon carcinogenesis in Wistar rats (13). We extracted concentrated ASP from shoots to evaluate its anti-tumorigenic effects in serous OC. This extract of ASP exhibited significant inhibition of cell viability and reduction of cell invasion, confirming that crude ASP extracts from shoots are rich in bioactive phytochemicals that inhibit the growth of cancer cells, including OC cells (12, 31).

Previous studies have proposed that ASP inhibits tumor cell growth by several mechanisms. ASP was initially reported to irreversibly inhibit DNA synthesis and cause cell growth inhibition in HL60 cells (24). Subsequently, it was found that inhibition of tumor cell growth by ASP was *via* induction of apoptosis and cell cycle arrest in cancer cells (29, 30). Asparanin A, a steroidal saponin extracted from ASP, induced p53-independent cell cycle G2 arrest and mitochondrial apoptosis in HepG2 cells (25). However, a more recent study found that treatment with Asparanin A, isolated from stems and spears of ASP, leads to induction of cell cycle G0/G1 arrest, activation of caspases pathway and reduction of mitochondrial membrane potential through the PI3K/AKT/mTOR pathway in endometrial cancer cells (30). In addition, the activation of TRAIL DR4/DR5 death receptor pathways has been recently described in ASP treated colon cancer cells, leading to the activation of caspase-8 and caspase-3 (13). In the current study, we found that ASP caused cell cycle G1 arrest, reduced mitochondrial membrane potential and activated caspase 3, 8 and 9, accompanied by a decrease in BLC-2 and MCL-1. Application of Z-VAD partially abrogated ASP’s effects on apoptosis and inhibition of cell proliferation. Given that BCL-2 family members control mitochondrial outer membrane permeabilization and trigger apoptosis through Bax/Bcl-2 ratio and caspase activation (32), our results suggest that inhibition of cell growth by ASP mainly depends on the extrinsic and intrinsic apoptotic pathways, in which apoptotic stimuli induce ROS and reduce mitochondrial membrane potential, which triggers cleavage of caspase 8 and 9 in apoptosomes and subsequent activation of the downstream executioner caspase-3, leading to inhibition of cell growth in ovarian cancer.

Metastasis of serous ovarian carcinomas involves detachment of tumor cells from the primary tumor site as well as direct extension to sites that are proximal to the primary tumor through the process of EMT which allows anchoring and extravagating cancer cells to regain epithelial features and proliferate into the tumor (33, 34). ASP has been documented to have inhibitory effects on the invasion of breast cancer cells in a dose-dependent manner by targeting the Rho GTPase signaling pathway (12). Treatment with polysaccharide from ASP in an orthotopic hepatocellular carcinoma model of Wistar rats significantly reduced CD34 and VEGF expression in tumor tissues, indicating an anti-angiogenic function for ASP *in vivo* (29). In line with these results, we observed that ASP treatment inhibited cell adhesion and invasion in a dose-dependent manner in serous OC cells and reduced VEGF levels in the serum and ovarian tumors of KpB mice. Thus, the underlying mechanisms by which ASP reduces invasion in OC cells may be related to ASP’s effects on the regulation of EMT and angiogenesis.

The combination of natural products with conventional therapies is an attractive strategy for the prevention and treatment of cancer, as many natural products are well-tolerated in patients (9, 35). Because ASP was efficacious as a single agent against multiple types of cancer in pre-clinical models, we further explored combining ASP with paclitaxel and the resulting anti-proliferative effects of this combination in OC cells. It has been reported that de-proteinized asparagus polysaccharide significantly potentiated the anti-tumorgenic effects of mitomycin in hepatocellular carcinoma cells and a mouse xenograft model of hepatocellular carcinoma (11). Similarly, our results found that the combination of low dose ASP and paclitaxel was synergistic in inhibiting proliferation in the OVCAR5 and SKOV3 cell lines as tested by MTT assay. Although more evidence is needed to better understand the underlying mechanism of action for the synergistic cytotoxicity of this combination, these results support further investigation of the combination treatment of ASP and paclitaxel in OC *in vivo*.

In conclusion, growing evidence suggests that ASP may be a novel dietary agent for the prevention and treatment of cancer, in addition to its potential overall health benefits. In addition to saponins and polysaccharides, we still have little understanding of the anticancer activity of other components of ASP and their underlying biochemical pathways. This study is the first to show that ASP is a potent inhibitor of cell proliferation and tumor growth in OC cells and the KpB high grade serous OC mouse model, under both obese and lean conditions. Thus, future studies are warranted to continue to explore ASP as a promising and well-tolerated dietary intervention as well as adjunct treatment in highly lethal ovarian cancer.

## Data Availability Statement 

The original contributions presented in the study are included in the article/**Supplementary Material**. Further inquiries can be directed to the corresponding authors.

## Author Contributions 

Conceptualization: VB-J and CZ. Methodology and experimental design: GX, WK, ZF, YF, YY, WS, LHC, TH, and CZ. Data collection and interpretation: GX, WK, SAS and A-QT. ASP provider: LZ. Drafted manuscript: CZ and VB-J. All authors contributed to the article and approved the submitted version.

## Conflict of Interest

Author LZ was employed by company Shandong Juxinyuan Agricultural Technology Co, Ltd.

The remaining authors declare that the research was conducted in the absence of any commercial or financial relationships that could be construed as a potential conflict of interest.
