# Supplementary material for: Asparagus officinalis Exhibits Anti-Tumorigenic and Anti-Metastatic Effects in Ovarian Cancer
Source: Front Oncol. 2021 Jul 14;11:688461. doi: 10.3389/fonc.2021.688461 (PMC8317209; doi:10.3389/fonc.2021.688461)
Supplement: Supplementary file 1 [file DataSheet_1.pdf]

荷泽巨鑫源食品有限公司

○ **HEZEJUXIN YUAN FOOD CO., LTD**

Inspection Report

Name:Heze Juxinyuan Food CO., LTD NO.JXY-YP-2-180501

Sample	Condensed Asparagus Juice	Amount	200Kg/Bucket
Date of production	2018．4．16	Date of detection	2018.4.25
		Basis	GB/T 31121

Result

NO.	Project	Unit	Standard	Result	Decision
1	subjective inspection	/	Color: even and milky<br>Odor: with asparagus unique odor, peculiar smell.<br>Impurities: No foreign impurities visible to the naked eye.	Normal	Qualified
2	Net content	Kg	About 200	200	Match
3	Soluble olid	%	60-68	62	Qualified
4	PH	/	3. 6-5. 6	4. 48	Qualified
5	Total bacterial count	cfu/ ml	W100	11	Match
6	Coliform	MPN/100 ml	W3	0	Match
7	Mycete	cfu/ ml	W10	2	Match
8	Accharomycetes	cfu/ m	<10	3	Match
9	Salmonella	/25g	n=5, c=0, m=0	No Detection	Qualified
10	Staphylococcus aureus	Cfu/ml	n=5, c=l, m=100, M=1000	<10/<10/<10/<10/<10	Qualified
11	Benzoic acid and sodium benzoate （calculated as benzoic acid）	g/kg	W1.0	No Detection	Qualified
12	sulfur dioxide	g/kg	W0. 05	0. 02	Qualified
13	Sorbic acid and potassium salt （calculated as Sorbic acid）	g/kg	W2.0	No Detection	Qualified
14	Saccharin sodium (calculated as saccharin)）	g/kg	No Detection	No Detection	Qualified
15	Acesulfame potassium	g/kg	W0. 3	No Detection	Qualified
16	Sodium cyclamate (calculated as cyclohexyl sulfamic acid)	g/kg	W0. 65	No Detection	Qualified

17	Tartrazine aluminum lake （calculated as Tartrazine）	g/kg	W0.1	No Detection	Qualified
18	Sunset yellow aluminum lake （calculated as Sunset yellow）	g/kg	W0.1	No Detection	Qualified
19	Brilliant blue aluminum lake（calculated as Brilliant blue）	g/kg	W0. 025	No Detection	Qualified
20	Poncean 4R Aluminum Lake （calculated as Poncean）	g/kg	WO. 05	No Detection	Qualified
21	Amaranth aluminum lake（calculated as Amaranth）	g/kg	W0. 05	No Detection	Qualified
22	total arsenic （As）	mg/kg	No Detection	No Detection	Qualified
23	lead （Pb）	mg/L	WO. 05	No Detection	Qualified
24	cadmium （Cd）	mg/kg	W0.5	No Detection	Qualified
25	iron	mg/L	W15	5.0	Qualified
26	copper	mg/L	W5	3.9	Qualified
27	tin	mg/L	200	<20.0	Qualified
28	zinc	mg/L	W5	1.5	Qualified
29	Hexachlorocyclohex-ane	mg/kg	WO. 2	No Detection	Qualified
30	Chlorophenothane	mg/kg	W0. 2	No Detection	Qualified
31	Dicofol	mg/kg	W1.0	No Detection	Qualified
32	Fenvalerate	mg/kg	W0. 5	No Detection	Qualified
33	Dichlorvos	mg/kg	WO. 1	No Detection	Qualified
34	Dimethoate	mg/kg	WO. 2	No Detection	Qualified
35	Chlorpyrifos	mg/kg	W0.2	No Detection	Qualified
36	Carbofuran	mg/kg	WO. 1	No Detection	Qualified
37	Triazophos	mg/kg	W0.2	No Detection	Qualified
39	Acephate	mg/kg	WO. 1	No Detection	Qualified
40	Fenitrothion	mg/kg	W0.5	No Detection	Qualified
41	Sign	/	GB7718-2011	Meet the Standard	Qualified
Result	**The inspected items of this product are qualified.**<br>Date 5/5/2018				

Auditor：Zien Li

Chief inspector：Yang Ma
