# Supplementary material for: Asparagus officinalis Exhibits Anti-Tumorigenic and Anti-Metastatic Effects in Ovarian Cancer
Source: Front Oncol. 2021 Jul 14;11:688461. doi: 10.3389/fonc.2021.688461 (PMC8317209; doi:10.3389/fonc.2021.688461)
Supplement: Supplementary file 2 [file DataSheet_2.pdf]

Supplemental table 2. Pesticide test report*

Number	Project	Unit	Standard	Result	Decision
29	Hexachlorocyclohexane	mg/kg	≤0.2	No Detection	Qualified
30	Chlorophenothane	mg/kg	≤0.2	No Detection	Qualified
31	Dicofol	mg/kg	≤1.0	No Detection	Qualified
32	Fenvalerate	mg/kg	≤0.5	No Detection	Qualified
33	Dichlorvos	mg/kg	≤0.1	No Detection	Qualified
34	Dimethoate	mg/kg	≤0.2	No Detection	Qualified
35	Chlorpyrifos	mg/kg	≤0.2	No Detection	Qualified
36	Carbofuran	mg/kg	≤0.1	No Detection	Qualified
37	Triazophos	mg/kg	≤0.2	No Detection	Qualified
39	Acephate	mg/kg	≤0.1	No Detection	Qualified
40	Fenitrothion	mg/kg	≤0.5	No Detection	Qualified

* This table is a part of translation from Supplemental table 1, and only shows the results of pesticide in ASP.
